# Chitosan Sponges as Next-Generation Biomaterials for Dental Tissue Engineering and Periodontal Regeneration

**DOI:** 10.3390/pharmaceutics17121622

**Published:** 2025-12-17

**Authors:** Magdalena Paczkowska-Walendowska, Maciej Kulawik, Jakub Kwiatek, Dimitrios Bikiaris, Judyta Cielecka-Piontek

**Affiliations:** 1 Department of Pharmacognosy and Biomaterials, Poznan University of Medical Sciences, 60-806 Poznan, Poland; maciej.kulawik@student.ump.edu.pl (M.K.); jpiontek@ump.edu.pl (J.C.-P.); 2 Science-Bridge Sp. z o.o., Chociszewskiego 24/8, 60-258 Poznan, Poland; 3 Kwiatek Dental Clinic Sp. z o.o., Kordeckiego 22, 60-144 Poznan, Poland; jakubkwiatek@klinikakwiatek.pl; 4 Laboratory of Polymer Chemistry and Technology, Department of Chemistry, Aristotle University of Thessaloniki, 54124 Thessaloniki, Greece; dbic@chem.auth.gr

**Keywords:** chitosan, sponges, stomatology, tissue engineering, periodontitis, bone regeneration, biomedical applications

## Abstract

Chitosan, a naturally derived polysaccharide obtained by chitin deacetylation, has attracted considerable attention in dentistry as a multifunctional biomaterial owing to its excellent biocompatibility, biodegradability, and tunable physicochemical properties. This narrative review provides an up-to-date overview of the use of chitosan-based sponges in dental tissue engineering, bone regeneration, post-extraction wound management, and periodontal therapy. Chitosan sponges, characterized by high porosity, flexibility, and superior absorbency, serve as effective wound dressings, drug delivery carriers, and scaffolds that promote cell proliferation and tissue regeneration. Their intrinsic antibacterial, antifungal, hemostatic, and immunomodulatory properties further enhance their therapeutic value in managing complex oral conditions. In periodontal treatment, they enable localized drug delivery and support soft and hard tissue healing, while in post-extraction care, they aid hemostasis and reduce complications such as alveolar osteitis. Moreover, their osteoconductive and osteoinductive potential positions them as promising materials for alveolar bone repair and implantology. Chemical modification of chitosan and the incorporation of bioactive compounds allow customization of sponge formulations to meet specific clinical needs. Despite encouraging preclinical findings, challenges remain due to variability in chitosan sources, differences in the degree of deacetylation, and limited clinical validation. This review highlights the potential of chitosan sponges as innovative tools in regenerative dentistry and underscores the need for further standardization, mechanistic studies, and long-term clinical trials to ensure their safe and effective translation into dental practice. Moreover, the broad clinical applications of chitosan sponges beyond dentistry confirm their potential as a universal biomaterial platform in regenerative medicine.

## 1. Introduction

The rapid advancement of biomaterials science in recent decades has profoundly transformed modern dentistry, providing new opportunities for the regeneration of soft and hard oral tissues, controlled drug delivery, and minimally invasive therapeutic strategies [1].

Given the increasing clinical demand for effective and biologically safe materials in oral surgery and periodontology, understanding the broader health and economic context in which these biomaterials are applied is essential. The prevalence of oral diseases and the frequency of surgical interventions highlight the need for accessible, cost-effective solutions capable of improving healing outcomes and reducing postoperative complications. Dental and oral diseases impose a substantial global economic burden, with the worldwide direct costs associated with oral conditions estimated at approximately US$ 387 billion in 2019, representing one of the highest categories of noncommunicable disease expenditures [2]. Historic analyses have similarly reported global dental treatment spending at nearly US$ 300 billion, accounting for around 4–5% of total healthcare costs [3]. In Europe, oral diseases remain highly prevalent, affecting nearly half of the adult population and driving continuous demand for surgical interventions, including tooth extractions, periodontological procedures, and implant-related treatments [4]. As a result, the need for efficient, affordable, and biologically safe biomaterials continues to grow, underscoring the relevance of developing next-generation wound-care materials.

Among naturally derived polymers, chitosan, a linear polysaccharide obtained through the deacetylation of chitin, has attracted considerable attention as a multifunctional material with a broad range of biomedical applications [5]. Its excellent biocompatibility, biodegradability, mucoadhesive properties, and ability to be chemically modified make it an ideal candidate for use in oral environments, which are characterized by constant exposure to mechanical stress, microbial flora, and dynamic pH conditions [6,7]. In dentistry, chitosan has been incorporated into various formulations, including films, hydrogels, nanoparticles, membranes, and scaffolds, owing to its capacity to promote wound healing and support tissue regeneration. However, among these, chitosan-based sponges represent a particularly promising form due to their high porosity, flexibility, and exceptional absorbency [8]. These properties enable sponges to function as effective wound dressings, hemostatic agents, drug delivery systems, and three-dimensional scaffolds that mimic the extracellular matrix and facilitate cell adhesion, proliferation, and differentiation. Their intrinsic antibacterial, antifungal, hemostatic, and immunomodulatory activities further enhance their therapeutic potential in managing complex oral diseases such as periodontitis, alveolar bone loss, and post-extraction complications [5,9].

The oral cavity presents unique biological and mechanical challenges that demand biomaterials capable of integrating with both soft and hard tissues. The oral environment is characterized by high variability in pH, a constant flow of saliva, and the presence of a complex microbiome, all of which significantly affect the stability and biocompatibility of applied biomaterials [10]. An additional challenge is the continuous impact of mechanical forces associated with chewing, speaking, and temperature fluctuations, which may lead to material degradation or loss of adhesive properties. The porous structure and modifiable surface chemistry of chitosan sponges allow for the incorporation of bioactive compounds, growth factors, and nanoparticles, creating multifunctional composite systems with tailored release profiles and enhanced bioactivity [11]. Despite the growing number of experimental and preclinical studies, the current literature on chitosan sponges in dental applications remains fragmented and lacks a comprehensive synthesis. There is still limited understanding of how parameters such as molecular weight, degree of deacetylation, crosslinking method, and additive composition influence their physicochemical behavior, degradation kinetics, and biological performance.

This review presents the first comprehensive and integrated analysis of chitosan-based sponges specifically in the context of dentistry. It discusses their fabrication methods, physicochemical and biological properties, and their applications in periodontal therapy, post-extraction management, pulp and bone regeneration. The innovative aspect of this work lies in correlating the material engineering perspective (e.g., structural design, crosslinking chemistry, and functional modification) with clinical utility, providing a translational framework that bridges laboratory findings and dental practice. Moreover, this paper highlights emerging strategies that combine chitosan with bioactive molecules, nanoparticles, and growth factors to create next-generation, smart sponges capable of targeted action, controlled degradation, and enhanced regenerative potential. By addressing the current research gaps and identifying future directions, this review aims to stimulate further development of standardized, clinically validated chitosan-based sponges as innovative biomaterials for regenerative dentistry.

## 2. Methodology of the Review

**Figure 1:**
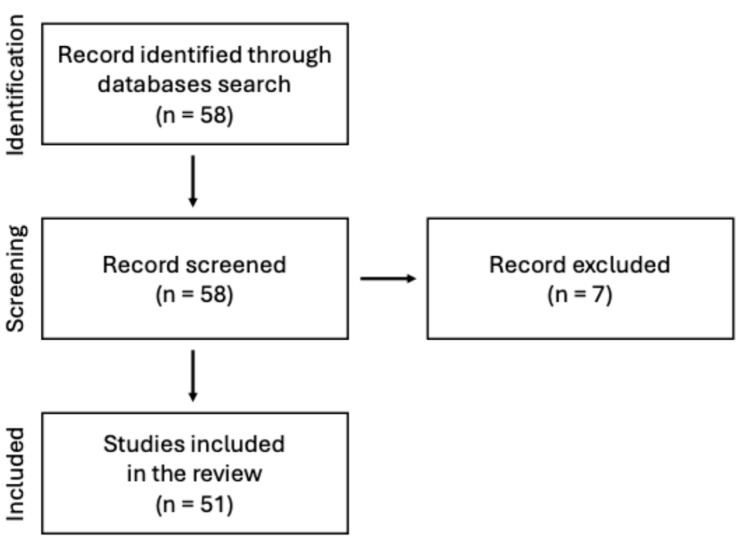
Search strategy presented on the flow diagram.

This manuscript presents a narrative review of the current state of knowledge on the use of chitosan-based sponges in dental applications. A comprehensive search of scientific literature was conducted using PubMed database. The keyword sequence used to search the database was the following: (“chitosan”) AND (“sponge” OR “sponges”) AND (“dentistry” OR “oral surgery” OR “dental” OR “stomatology”) AND (“periodontitis” OR “wound healing” OR “bone”). The search strategy focused on original research and review articles published in peer-reviewed journals, with particular emphasis on chitosan and its application in the form of sponges within dentistry, especially in areas such as periodontal disease, wound healing and tissue regeneration. Abstracts were screened to assess the relevance of each article. Inclusion criteria were limited to English-language papers published before mid-2025. Figure 1 illustrates the adopted literature search strategy.

This review is limited using selected database and the exclusion of non-English publications. Moreover, variations in chitosan source, degree of deacetylation, molecular weight, and crosslinking methods complicate direct comparisons across studies. Many clinical applications are still at an experimental or early validation stage, and no meta-analysis was performed.

## 3. Chitosan

### 3.1. Physicochemical Properties

**Figure 2:**
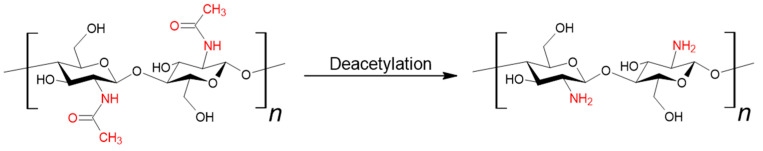
Synthesis of chitosan from chitin.

Chitin (β-(1-4)-poly-N-acetyl-D-glucosamine) is one of the most abundant naturally occurring polysaccharides, ranking second only to cellulose. It is the primary component of the exoskeletons of crustaceans, crabs, and shrimps. It is also a structural element of the fungal cell wall [12,13,14]. Chitosan can be obtained from chitin through deacetylation (see Figure 2). This process is most carried out using a strong base, and less frequently through enzymatic methods or the use of ionic liquids. The key parameter distinguishing these two polymers is the degree of deacetylation; a value equal to or greater than 50% qualifies the material as chitosan [15].

Chitosan is a linear polysaccharide composed of N-acetyl-2-amino-2-deoxy-D-glucose (glucosamine, GlcN) and 2-amino-2-deoxy-D-glucose (N-acetyl-glucosamine, GlcNAc) units linked by β-(1-4) glycosidic bonds [16,17,18,19]. The molecular weight of the polysaccharide can range from 50 to 1000 kDa. This parameter depends on the source and processing method [20]. Chitosan is classified based on molecular weight into three groups: high molecular weight (HMW) above 700 kDa, medium molecular weight (MMW) ranging from 150 to 700 kDa, and low molecular weight chitosan (LMW) below 150 kDa [21]. This polymer is insoluble in water (pH = 7) and most liquid organic solvents. Such media include lactic, acetic, formic, and citric acids, as well as 10-camphorsulfonic acid, p-toluenesulfonic acid, and dimethyl sulfoxide [22]. In acidic water environments, the amino groups (-NH_2_) become protonated, while under basic and physiological conditions, these groups are deprotonated, causing the polymer to precipitate [23]. Solubility is also influenced by the molecular weight of the polymer; as the molecular weight decreases, solubility increases (Table 1) [22]. The presence of a positive charge enhances binding capacity, thereby supporting effective drug penetration and enabling strong and durable attachment, a characteristic feature of chitosan [24].

**Figure 3:**
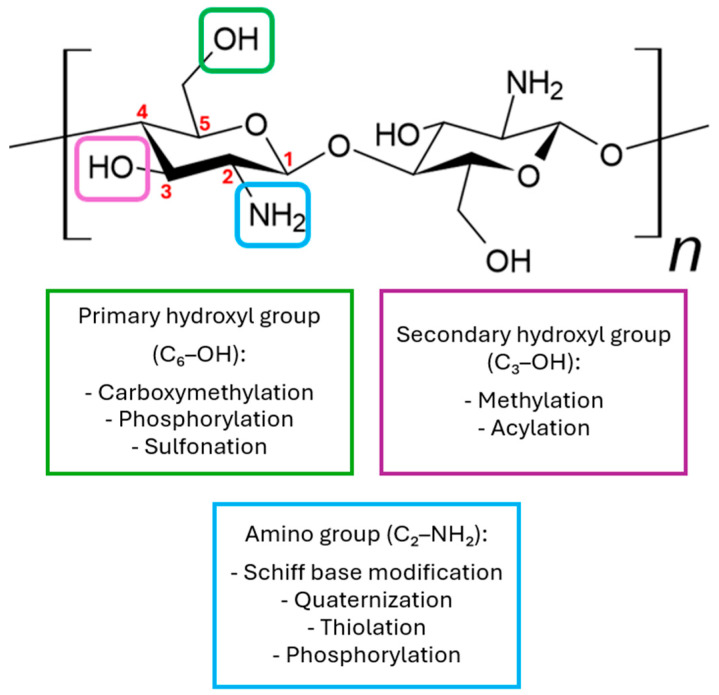
Examples of chemical modifications of chitosan.

Due to the presence of -NH_2_ and -OH groups, this compound can be chemically modified [25,26,27]. In its natural form, chitosan possesses three functional groups located at different positions: C6-OH, C3-OH, and C2-NH_2_ (Figure 3). Among these, the C2-NH_2_ and C6-OH groups are readily accessible for chemical modification, while modification of the C3-OH group is less favorable due to significant steric hindrance. Amino groups are most modified, as the C2-NH_2_ group exhibits high reactivity, which greatly facilitates substitution reactions. Although amino groups show higher reactivity toward nucleophiles, both OH and NH_2_ groups can also participate in electrophilic reactions with reagents such as acyl chlorides, acids, or alkanes, potentially leading to non-selective functionalization of these groups [22,28]. The main derivatives used in medicine are N,N,N-trimethyl chitosan, N,O-carboxymethyl chitosan, and O-carboxymethyl-N,N,N-trimethyl chitosan [23]. Various chemical modification techniques are used to alter the physicochemical properties of chitosan, such as sulfonation, methylation, and acylation. Additionally, processes like acylation, thiolation, phosphorylation, and succinylation are also employed to modify these properties [24].

Chitosan can independently form physical gels through non-covalent interactions such as hydrogen bonding, hydrophobic interactions, and electrostatic forces. It can also gel in the presence of anionic macromolecules (e.g., hyaluronic acid, alginate) by forming polyelectrolyte complexes. In situ gelation of chitosan refers to the transition from a liquid to a gel state in response to environmental changes such as pH, temperature, or the presence of ions. This property is particularly useful in ophthalmic applications [29].

### 3.2. Biological Properties

Chitosan exhibits high biocompatibility, making it an attractive material for biomedical applications, particularly as a drug delivery carrier [30,31]. In both in vitro and in vivo studies chitosan and its delivery systems have rarely been shown to cause significant cytotoxic, genotoxic, or inflammatory effects. When such effects do occur, they are generally dependent on the dose, form (solution vs. nanoparticles), degree of deacetylation, molecular weight, and the pH of the surrounding environment [30,32,33].

The polycationic nature of this polymer promotes the formation of complexes with oppositely charged molecules, facilitating its adhesion to mucous membranes [24,34]. Protonated amino groups enable electrostatic interactions with the anionic groups present in mucus, such as sialic and sulfonic acids [35,36]. The mucoadhesive strength of chitosan increases with higher molecular weight and a greater degree of deacetylation, as these characteristics enhance the number of available amino groups. On the other hand, excessive crosslinking of the chitosan structure may reduce its ability to bind to mucus. There are also modified forms of chitosan that demonstrate even stronger mucoadhesive properties. Trimethyl chitosan possesses a higher positive charge and better solubility in neutral and alkaline environments, which improves its ability to adhere to mucosal membranes. Carboxymethyl chitosan contains both amino and carboxyl groups, allowing it to adhere to mucus across a wide pH range, both acidic and alkaline. Additionally, the presence of thiol groups enables the formation of stable bonds with cysteine residues in mucin glycoproteins, further enhancing the mucoadhesive effect [36].

Chitosan is characterized by high bioabsorbability, which means it can be effectively absorbed and degraded in the human body. The ability of chitosan to be bioabsorbed is closely related to its chemical structure, particularly the degree of deacetylation and molecular weight [37]. Although no single universal threshold has been established, studies consistently show that chitosan with a DD% above 70–75% undergoes noticeably faster bioabsorption due to increased accessibility of glycosidic bonds to lysozyme-mediated degradation. Even more pronounced effects occur when DD% exceeds 85%, where the polymer becomes more cationic and enzymatically susceptible, resulting in accelerated resorption. In general, higher DD% values correlate with faster bioabsorption, although the precise degradation rate is also influenced by molecular weight, crystallinity, and the degree of crosslinking in the final material [25]. The ability to degrade chitosan is exhibited, among others, by lysozyme [38]. As a strategy to control degradation and enhance antibacterial properties, the incorporation of this enzyme into the structure of the polymer formulation is proposed [39,40].

Chitosan exhibits antibacterial and antifungal activity. Its antimicrobial properties depend on its structure, physicochemical characteristics, environmental conditions, and the presence of reactive hydroxyl groups. The action of chitosan can be divided into extracellular and intracellular effects [41]. HMW chitosan acts primarily extracellularly by forming a barrier on the surface of microbial cells, which disrupts the exchange of nutrients and chelates metal ions essential for cell growth. LMW chitosan can penetrate microbial cells, interacting with internal structures such as DNA and RNA, which leads to the inhibition of protein synthesis and mitochondrial functions [42]. A higher degree of deacetylation enhances the electrostatic binding of the polymer to the negatively charged bacterial cell membrane. At physiological pH (~7.4), chitosan loses its positive charge, becomes insoluble, and acts by forming a physical barrier on the surface of bacterial cells [43]. The type of bacteria is another factor influencing the effectiveness of chitosan. Gram-positive bacteria, due to the absence of an outer membrane, are often more susceptible to chitosan. However, some studies indicate that Gram-negative bacteria, despite having an additional barrier, may be more vulnerable to its action due to their higher surface hydrophilicity and greater density of negative charge [43,44]. Activity against Gram-negative bacteria is of particular importance in the context of the oral cavity, as these microorganisms often play a key role in the development of infections and periodontal inflammation (the red bacterial complex and, to some extent, the orange complex) [45]. The source of chitosan also plays a significant role here due to differences in the degree of deacetylation [46]. Chemical modifications of chitosan enhance its solubility and antibacterial activity. Derivatives with permanent positive charges, such as trimethylated chitosan salts, are particularly effective, demonstrating strong antimicrobial effects [47]. Chitosan does not always exhibit antibacterial activity; its effectiveness depends on its concentration and molecular weight. As demonstrated in studies, at low concentrations, certain forms of chitosan may even stimulate the growth of *E. coli* instead of inhibiting it. This indicates that chitosan should not be considered a universal antimicrobial agent without prior adjustment of its physicochemical parameters [48]. Interestingly, recent research has also highlighted chitosan’s potential as a prebiotic substance. For example, cricket-derived chitosan has been shown to significantly promote the growth of beneficial probiotic bacteria such as *L. fermentum*, *L. acidophilus* and *B. adolescentis*. These bacteria were able to utilize chitosan as a fermentable substrate, leading to increased biomass and a reduction in pH, which in turn contributed to the suppression of pathogenic species like *Salmonella typhi*. This dual role, supporting probiotic growth while inhibiting pathogens, suggests that, under optimized conditions, chitosan may serve not only as an antimicrobial but also as a functional prebiotic ingredient in gut health interventions [49].

Chitosan acts as a hemostatic agent through several mechanisms that are independent of the classical blood coagulation pathway [50]. Its positively charged molecules interact electrostatically with the negatively charged membranes of erythrocytes, leading to rapid clot formation. Additionally, chitosan promotes platelet adhesion and aggregation and can bind to plasma proteins and clotting factors, enhancing the coagulation process. As a linear polysaccharide, it forms a network structure that facilitates the formation of stable clots. Due to these properties, chitosan is an effective material for controlling bleeding, especially in challenging clinical conditions [51].

## 4. Preparation of Chitosan Sponges

**Figure 4:**
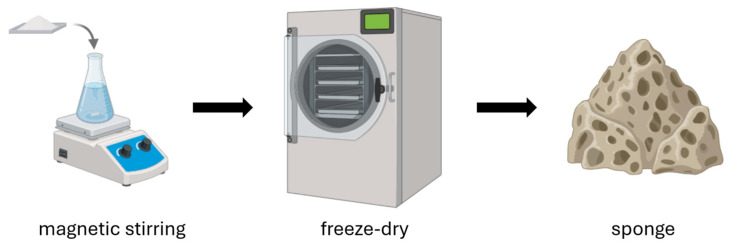
Preparation of chitosan sponges.

Chitosan sponges are advanced bioactive materials that, thanks to their porous structure and the biological properties of chitosan, are used in the production of wound dressings. The manufacturing process involves several key steps (Figure 4). The first step is preparing a chitosan solution. Chitosan is dissolved in an acid solution (most commonly acetic acid), forming a homogeneous hydrogel [52,53]. Depending on the intended application, other functional components can be added to the solution, such as silver nanoparticles, synthetic or natural polymers, and biologically active substances. Next, an optional foaming process can be performed to impart a porous structure to the material [52,54]. There are three main foaming methods: mechanical (intense mixing to introduce air), physical (using pore-forming agents such as sodium sulfate or gases like CO_2_), and chemical (chemical reactions that generate gases, e.g., carbonate decomposition) [53]. Then, the hydrogel is frozen and lyophilized (freeze-dried), allowing the removal of water and resulting in a stable, lightweight, porous sponge [52,53,54,55]. Sometimes the freezing step is omitted, and the chitosan solution is subjected directly to lyophilization [56]. The method used to produce chitosan sponges can also be applied to the fabrication of porous microspheres. During their production, it has been observed that direct lyophilization without freezing, as well as the freezing temperature of the chitosan solution, influence the structure of the resulting scaffolds [57]. However, it is important to consider that the addition of various auxiliary substances can also influence the final structure of the resulting scaffold.

Finished chitosan sponges are characterized by high porosity, excellent absorbency, flexibility, and biocompatibility [53]. Due to their high liquid absorption capacity, chitosan sponges are also proposed as effective absorbents. The production of water-insoluble sponges can be achieved by washing them in an alkaline solution, followed by rinsing with water and drying [58].

Besides freeze-drying, several alternative methods have been developed to tailor the structure and properties of chitosan sponges. The foaming technique introduces gas bubbles mechanically or chemically, producing lightweight, porous sponges suitable for hemostatic and drug delivery applications, although with lower mechanical strength [59]. Crosslinking and gelation, using agents such as genipin, glutaraldehyde, or tripolyphosphate, improve stability, water resistance, and control degradation rate, making the sponges more suitable for bone regeneration [60]. The solvent casting and particulate leaching method employs porogens such as salt or sugar to create uniform, interconnected pores after leaching, resulting in scaffolds with adjustable pore size and good mechanical integrity. In addition, hybrid approaches, including electrospinning and freeze-gelation, enable the fabrication of multilayer or fibrous chitosan–polymer composites that better mimic the extracellular matrix. These methods collectively expand the versatility of chitosan sponges, allowing their properties to be optimized for specific dental and regenerative applications (Table 2).

Among the available fabrication strategies, methods that combine freeze-drying with crosslinking or the formation of chitosan–inorganic composites (e.g., hydroxyapatite) demonstrate the highest mechanical stability, particularly in load-bearing oral environments. Crosslinking agents such as genipin or tripolyphosphate introduce covalent or ionic bonds that reinforce the polymer network, reduce collapse upon hydration, and enhance compressive strength. Similarly, incorporating mineral phases increases stiffness and better mimics the structural characteristics of natural bone, making such composite scaffolds more resistant to deformation under masticatory forces. As a result, crosslinked and composite freeze-dried chitosan scaffolds are currently considered the most mechanically robust configurations for dental and regenerative applications requiring enhanced structural durability.

## 5. Chitosan Sponges in Stomatology

**Figure 5:**
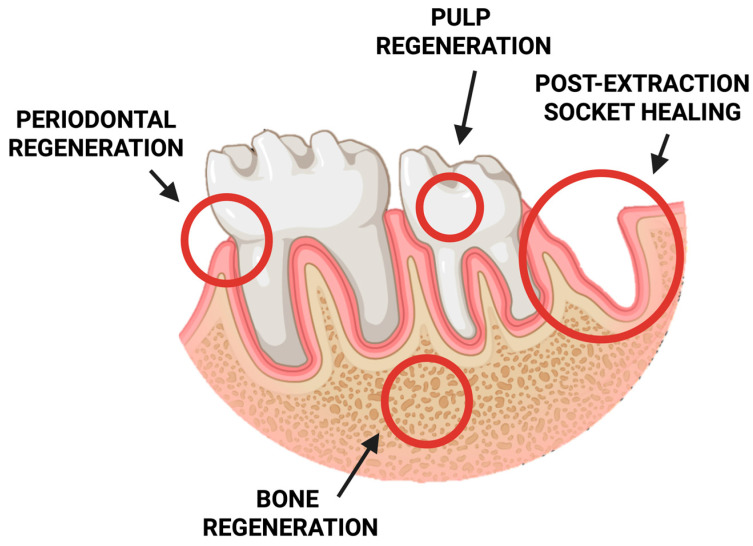
Schematic representation of potential clinical applications of chitosan-based sponges in dentistry.

In recent years, chitosan-based sponges have gained significant attention in stomatology due to their exceptional biocompatibility, biodegradability, and multifunctional properties. Their highly porous, three-dimensional structure provides an ideal environment for tissue regeneration, drug delivery, and hemostasis within the complex oral environment. These features make chitosan sponges promising materials for a wide range of dental applications, including wound healing, periodontal therapy, pulp and bone regeneration, and post-extraction management (Figure 5).

### 5.1. Post-Extraction Treatments

**Figure 6:**
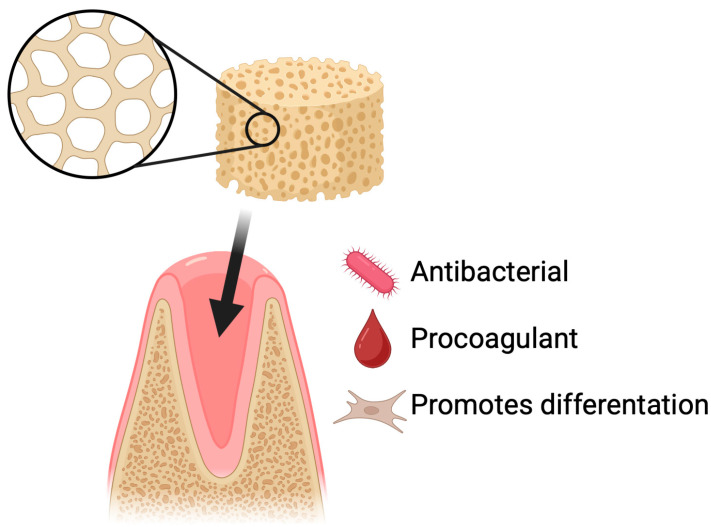
Dry socket prevention using chitosan-based sponges. Application of chitosan sponges to extraction sockets supports rapid clot stabilization, protection of the wound site, and reduction in postoperative complications. Their porous and mucoadhesive structure promotes blood absorption, formation of a stable fibrin network, and physical shielding of the socket from mechanical disruption and bacterial contamination. By maintaining a moist environment and providing intrinsic antibacterial and hemostatic properties, chitosan sponges decrease the risk of alveolar osteitis and facilitate faster tissue healing following tooth extraction.

Dry socket is the most common complication following tooth extraction and is also one of the best-studied post-extraction complications [61,62]. Prevention is primarily based on physical protection of the clot (including proper wound suturing) and chemical protection through the use of antiseptics or topical agents that support healing [63]. The use of various biomaterials, such as sponges, membranes, hydrogels, nanofibers, and particles, supports the healing of the post-extraction socket. These materials can serve as carriers for bioactive substances and cells, promote tissue regeneration, and prevent infections [64]. One of the polymers that can support the regeneration of soft and hard tissues after tooth extraction is chitosan [65,66,67]. Dressings in the form of gelatin sponges are currently being tested on humans with good results [68]. However, there is little research on lyophilized chitosan sponges (Figure 6).

Innovative approaches to pain management and hemostasis after deciduous tooth extraction are key in pediatric dentistry. Thakkar et al. compared the effectiveness of low-level laser therapy (LLLT) and chitosan sponge in controlling pain and bleeding after extraction of deciduous molars. The randomized crossover clinical trial involved 29 children (87 teeth), divided into three groups with a different order of intervention: gauze (control), LLLT (940 nm diode laser, 0.8 W, 1 min) and chitosan (7 mm sponge placed in the alveolus). LLLT showed the lowest pain scores immediately after intervention and 24 h. There were no significant differences in hemostasis after 15 min between the groups, although LLLT and chitosan showed faster bleeding arrest after 10 min. Chitosan was preferred by children and parents due to its ease of application. The study underscores the superior efficacy of LLLT in pain control, but chitosan is an effective, cost-effective alternative, especially in hemostasis and patient acceptance, which may find application in the treatment of post-extraction complications in children [69].

To prevent the formation of dry socket, Deng et al. designed antibacterial sponges with sodium alginate as the main material. These sponges contained chitosan in the form of a quaternary ammonium salt as an additive. The composite ACQ sponges were obtained by combining sodium alginate, carboxymethyl starch, and chitosan modified with quaternary groups. The components were dissolved in water, then formed, cross-linked with calcium ions, and lyophilized. The resulting materials exhibited a porous structure, high fluid absorption capacity, and suitable water vapor permeability. In vitro studies demonstrated good cytocompatibility and low hemolysis, while biological tests confirmed effective inhibition of *E. coli*, *S. aureus*, and *C. albicans* growth. The ACQ sponges also showed strong procoagulant properties. In vivo tests, conducted both in a mouse liver injury model and after tooth extraction in miniature pigs, showed that the ACQ sponges had hemostatic properties by reducing bleeding time and volume. These results suggest that ACQ sponges could be a promising material for clinical post-extraction use, particularly in the prevention of dry socket [70].

In a study published by Yan et al., bio-multifunctional sponges based on calcium alginate were developed, incorporating chitosan/calcium phosphate-based microflowers and metronidazole, intended for the prevention of dry socket after tooth extraction. The sponge formulation was not based on chitosan; rather, chitosan was present only within the nanoparticles. The alginate-based sponges were obtained through calcium ion crosslinking and freeze-drying, which enabled the creation of a porous, hierarchical structure shaped to fit the tooth socket. The microflowers exhibited a three-dimensional, uniform morphology, enhancing their functional performance. The study results demonstrated that the sponges possessed excellent hemostatic, antibacterial, and osteogenic properties. In mechanical tests, the sponges showed high compressive strength in artificial saliva and blood, confirming their ability to effectively control bleeding. Antibacterial assays revealed significant inhibition of *S. aureus* and *E. coli*, attributed to the synergistic effect of metronidazole, which damages bacterial DNA, and chitosan, which disrupts bacterial membrane permeability. Cytotoxicity assessment using MC3T3-E1 cells confirmed the high biocompatibility of the sponges. Moreover, the sponges significantly enhanced alkaline phosphatase activity, a key marker of early osteogenesis, indicating their potential to promote osteoblast differentiation and bone regeneration. The inclusion of microflowers, rich in calcium and phosphorus, supported osteogenic processes by activating signaling pathways such as CaMK II, CREB, and ERK, without any negative interference from metronidazole. Overall, the alginate sponges demonstrated strong potential as an effective post-extraction socket-filling material, preventing dry socket due to their outstanding hemostatic, antibacterial, and osteogenic properties, making them a promising solution for clinical applications [71].

### 5.2. Oral Tissues Regeneration

Soft tissue regeneration in dentistry involves the restoration of gums, oral mucosa, or periodontal tissues that have been damaged by diseases, injuries, or surgical procedures. This process relies on the body’s natural healing abilities and can be supported by biomaterials [72,73]. The epithelium of the oral cavity behaves differently from human skin, which requires the use of distinct treatment strategies [74]. Tissue regeneration in dentistry can be supported by the use of appropriate stem cells, which have the ability to differentiate into the specific tissue being treated [75,76]. Tissue engineering is increasingly making use of scaffolds, whose role is to promote cell growth [77]. Chitosan is a biomaterial that can be used as a matrix for cell growth [78,79].

Sun et al. developed PCGS sponges (procyanidin-functionalized chitosan/gelatin sponges), which were obtained through a two-step process. First, composite chitosan–gelatin sponges (CGSs) were produced by freeze-drying an intensely mixed solution of chitosan and gelatin. Subsequently, these sponges were chemically crosslinked using a natural polyphenol, procyanidin. This process yielded functionalized PCGS sponges with a distinctly porous structure. It was shown that PCGS exhibited enhanced liquid absorption capacity (up to 4000% of their own weight), reduced surface charge, low hemolysis, and high antioxidant activity, with slightly reduced antibacterial activity compared to CGS. In vitro tests confirmed the hemostatic efficacy of PCGS. In vivo studies demonstrated that PCGS shortened bleeding time and reduced blood loss in a rat femoral artery injury model, as well as supported healing of infected post-extraction wounds in the oral cavity. Histological analysis showed reduced inflammatory infiltration and better tissue closure compared to treatment with adrenaline. These results indicate that PCGS sponges are a promising material for use as a multifunctional hemostatic dressing with antimicrobial and antioxidant properties [80].

Lu et al. designed a bilayer composite scaffold (Janus scaffold) consisting of a chitosan sponge and an electrospun PLGA/PCL membrane. The sponge was modified by adding chlorhexidine (CHX), epidermal growth factor (EGF), and basic fibroblast growth factor (bFGF), and then covalently bonded to the fibrous membrane using polydopamine. The preparation involved freezing the chitosan solution with additives, freeze-drying, and crosslinking, resulting in porous, flexible structures. The scaffold demonstrated good porosity, thermal stability, and appropriate elasticity. Antibacterial tests against *E. coli*, *S. aureus*, and *S. mutans* confirmed the effectiveness of the sponge. In vitro tests with rat fibroblasts analyzed proliferation, cell morphology, and apoptosis, indicating good biocompatibility and absence of cytotoxicity. A rat cranial bone defect model confirmed that the modified scaffold, especially those containing EGF, bFGF, and CHX, significantly accelerated regeneration of both soft and hard tissues. Histological observations revealed neovascularization and increased collagen synthesis. These results suggest that the proposed Janus scaffold is a promising clinical strategy to support simultaneous regeneration of soft and hard tissues in dental surgery [81].

#### 5.2.1. Dental-Pulp Regeneration

A noteworthy topic is the regeneration of dental pulp, which can be damaged by various factors such as bacteria, physical trauma, or temperature [82]. Root canal treatment, which involves the removal of the pulp, may result in the weakening of the tooth, which is also deprived of its ability to respond to sensory stimuli and to mount an immune response [83]. Methods aimed at pulp regeneration can spare the tooth from root canal treatment, thereby preventing it from becoming weakened.

A composite formulation composed of three biomaterials was presented by Loukelis et al. They developed a sponge based on κ-carrageenan, chitosan, and gelatin. The scaffolds were prepared by mixing polymer solutions, followed by freeze-drying and crosslinking with glutaraldehyde, which resulted in highly porous structures. The scaffolds exhibited high porosity and water absorption capacity. Biocompatibility was assessed using dental pulp stem cells, with PrestoBlue and live/dead staining confirming high cell viability and proliferation over a 14-day period. SEM analysis showed cell adhesion and pore infiltration by day 10. Odontogenic differentiation was evaluated through alkaline phosphatase activity, calcium deposition, and gene expression analysis (DSPP, ALP, RunX2) via qPCR. These results indicate that the scaffold serves as a promising biomimetic platform for dentin-pulp regeneration in dental tissue engineering. This kind of scaffold, combining natural polymers demonstrated clear regenerative potential [84].

Alghofaily et al. described scaffolds serving as antibiotic carriers based on chitosan and gelatin, which were prepared using the lyophilization method. Gamma irradiation was used as the sterilization method for the material. The sponges were loaded with Augmentin, a modified triple antibiotic paste (mTAP) containing clindamycin, amoxicillin or cefaclor instead of minocycline, as well as calcium hydroxide. The study demonstrated that chitosan–gelatin scaffolds soaked with Augmentin and mTAP at effectively eliminated *E. faecalis* biofilms, achieving a high percentage of dead bacterial cells. Augmentin at a lower concentration (0.1 mg/mL) most effectively supported the proliferation and adhesion of human mesenchymal stem cells, showing high biocompatibility. Scaffolds with calcium hydroxide showed limited antibacterial efficacy and weaker cell adhesion. All substances exhibited an initial burst release within the first hour, reaching nearly complete drug release after 7 days. The scaffolds maintained a porous structure conducive to tissue regeneration. These findings suggest that scaffolds loaded with Augmentin and mTAP are promising candidates for regenerative endodontic procedures [85].

#### 5.2.2. Periodontal Disease

**Figure 7:**
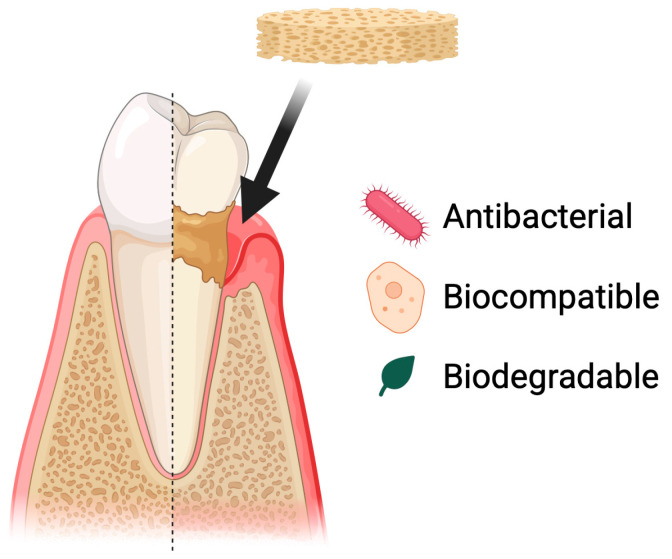
Use of chitosan-based sponges in periodontal diseases. Chitosan sponges act as local therapeutic carriers and regenerative scaffolds in periodontal defects. Their porous structure enables rapid blood absorption, clot stabilization, and infiltration of periodontal ligament and bone-forming cells. Due to strong mucoadhesion and controlled degradation, the sponges maintain prolonged retention in periodontal pockets, supporting sustained delivery of antibiotics, anti-inflammatory agents, or growth factors. This multifunctional activity contributes to infection control, modulation of inflammation, and promotion of new tissue formation within the periodontal environment.

Periodontal diseases include, among others, gingivitis and periodontitis. These conditions involve chronic inflammation of the tissues surrounding the teeth [86]. If left untreated, they are the leading cause of tooth loss in adults [87]. These conditions are caused by bacteria present in dental plaque, which leads to tissue recession. Poor oral hygiene and tobacco smoking are the main risk factors [88]. Periodontal diseases affect most of the population. This condition can negatively influence patients’ quality of life, partly due to esthetic concerns associated with tooth loss. Tooth loss or increased tooth mobility impairs normal oral function, leading to disturbances in chewing, articulation, and an acceleration of degenerative processes within the oral cavity. Moreover, periodontal diseases are an important risk factor for systemic conditions such as cardiovascular diseases, worsening of diabetes, and metabolic disorders, while chronic periodontal inflammation may modulate immune responses and intensify inflammatory processes throughout the body [89,90]. Treatment is based on a holistic approach that includes both prevention and therapeutic intervention. Regular dental check-ups and proper oral hygiene are of key importance, as they help maintain a balanced microbiome and prevent the development of inflammatory conditions [91]. Biomaterials, including chitosan, can be used as supportive agents in the treatment of periodontal diseases. They can serve as a basis for developing drug delivery systems, wound dressings, and scaffolds that support cell growth (Figure 7).

As a method for repairing soft and hard tissue defects in periodontal disease, Li et al. proposed a chitosan sponge supplemented with Transforming Growth Factor-β3 (TGF-β3). TGF-β3, which has a beneficial effect on tissue regeneration, was added to the lyophilized sponges. It also supports the maintenance of stem cell stemness and the formation of the extracellular matrix. The sponges exhibited high porosity, biocompatibility, and supported the growth of primary human periodontal ligament stem cells. TGF-β3 did not affect cell proliferation but promoted osteogenesis. COL I expression increased, while COL II and ALP levels decreased, and the p38 MAPK pathway was activated. The TGF-β3/CS sponges demonstrated potential for the repair of alveolar bone defects [92].

Chitosan sponges have also been studied as carriers for antibiotics. Shen et al. (2008) investigated the release of tetracycline from tripolyphosphate-crosslinked chitosan sponges for potential application in periodontology [93]. Four types of chitosan sponges were prepared: a control sponge, one with tetracycline (TC), one crosslinked with tripolyphosphate (TPP), and one both crosslinked and loaded with tetracycline (TPP-TC). The sponges were made using high-purity chitosan dissolved in a vitamin C solution, then formed by freezing, drying, and optionally crosslinking. TPP-TC sponges demonstrated prolonged tetracycline release for up to 11 days, with the highest concentration observed on day 9, while TC sponges degraded before day 9. The TPP-TC sponges maintained antibacterial activity for 11 days, forming zones of bacterial growth inhibition. TPP crosslinking increased sponge mass but reduced their thickness and diameter. The study suggests that TPP-TC sponges are suitable carriers for the controlled release of tetracycline, which could be beneficial in periodontal disease treatment, although further research is needed on the release mechanism and efficacy against periodontal pathogens [93].

The article published by Liao et al. describes a study on a bioactive, three-dimensional scaffold composed of β-tricalcium phosphate (β-TCP) and chitosan. The scaffolds were prepared using the lyophilization method, resulting in a uniform, porous structure. In vitro studies showed that the scaffold supported the adhesion, proliferation, and migration of human periodontal ligament cells more effectively than pure chitosan scaffolds. RT-PCR analysis revealed increased expression of BSP and CAP genes, which are associated with bone and cementum formation. In vivo studies, conducted after subcutaneous implantation in mice, demonstrated that the scaffolds were biocompatible, biodegradable, and capable of inducing blood vessel growth and the synthesis of proteins essential for hard tissue formation. These results suggest that the scaffold is a promising material for periodontal tissue regeneration, supporting the differentiation of cells toward osteoblastic and cementoblast phenotypes [94].

### 5.3. Bone Regeneration

**Figure 8:**
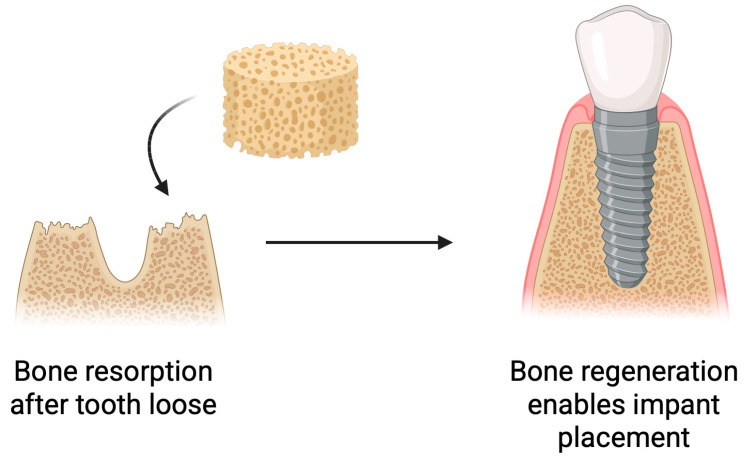
Use of chitosan-based sponges in bone regeneration. Schematic overview of the application of chitosan sponges as osteoconductive scaffolds supporting bone regeneration. The porous architecture facilitates rapid blood absorption, clot stabilization, and cellular infiltration, while the polymer matrix provides a temporary 3D framework for osteoblast adhesion and extracellular matrix deposition. Incorporation of bioactive molecules (e.g., antibiotics, growth factors, or nanoparticles) further enhances antibacterial performance, angiogenesis, and mineralized tissue formation. Such multifunctional sponges promote hemostasis, protect the surgical site, and gradually degrade as newly formed bone replaces the scaffold.

Following tooth loss, biological processes occur within the alveolar bone, leading to bone resorption and atrophy. This poses challenges for implant placement [95,96]. Insufficient bone volume negatively affects the long-term success of implantation and, in some cases, makes it impossible [97]. To prevent this or to restore the lost bone, various strategies are employed [98]. Bone reconstruction is a commonly used practice in implantology [99]. Scaffolds used for bone regrowth can release active substances that support its growth [100]. Current research indicates that the type of biomaterial used has an impact on bone regeneration [99,101]. One of the biomaterials used in bone tissue regeneration is chitosan. It can be applied both on its own and in combination with other materials [102,103,104]. Chitosan exhibits a range of properties that make it an attractive material for bone tissue engineering. It actively supports the bone regeneration process by stimulating osteoblast differentiation, increasing bone density, and accelerating fracture healing (Figure 8) [105].

In bone tissue engineering, the molecular characteristics of chitosan play a critical role in determining its mechanical stability, degradation profile, and biological performance. Chitosan used for bone scaffolds typically exhibits a molecular weight (MW) in the range of 100–400 kDa, which provides a balance between adequate structural integrity and processability during scaffold fabrication. Likewise, the degree of deacetylation (DD%) commonly falls between 70% and 95%, as higher deacetylation increases the density of free amino groups, improving cell adhesion, osteoconductivity, and enzymatic degradability. These parameter ranges enable chitosan scaffolds to support early osteogenic activity while maintaining a degradation rate compatible with the stages of bone remodeling. However, optimal values may vary depending on the scaffold architecture, crosslinking method, and specific clinical application, underscoring the need for standardized characterization protocols in future research.

In an eight-week preclinical study, Park et al. analyzed the effect of a chitosan-collagen sponge on periodontal tissue regeneration in surgically created one-wall bone defects in beagle dogs. The experiment included three groups: a control group (surgical procedure only), a buffer group (collagen sponge soaked in phosphate-buffered saline), and a test group (collagen sponge soaked in a 20 mg/mL chitosan solution). Although chitosan sponges were not used directly, the incorporation of chitosan into the scaffold makes this study particularly noteworthy. Histological and histometric analyses after eight weeks revealed that the chitosan group exhibited significantly reduced apical migration of the junctional epithelium, a desirable outcome that supports periodontal regeneration. This group also showed a markedly greater amount of newly formed cementum and alveolar bone compared to both the control and buffer groups. The cementum displayed features typical of active forms; it was thick, cellular, and often lined with cementoblasts. Additionally, perpendicular orientation of periodontal ligament fibers was observed in the area of new bone and cementum, indicating functional regeneration of the periodontium. New bone formed from the apical region toward the coronal direction, although its thickness was limited by the defect configuration, highlighting the importance of materials that help maintain regenerative space. In contrast to the chitosan group, the buffer and surgical control groups showed greater epithelial migration and less intense regenerative activity. While the collagen sponge alone did not induce osteogenesis, it served as a scaffold stabilizing the blood clot and supporting cellular colonization. Root resorption was superficial and present across all cases, an expected finding in experimental periodontal defect models [106].

Shinohara et al. investigated the regenerative potential of recombinant human bone morphogenetic protein-9 (rhBMP-9) in combination with chitosan and collagen sponges in a rat calvarial defect model. Collagen sponges combined with rhBMP-9 demonstrated the highest defect closure rate and the largest area of newly formed bone. They provided better structural maintenance of the defect due to slower biodegradation, which supported the controlled release of rhBMP-9. This led to intense trabecular bone formation with numerous osteocytes and blood vessels. Chitosan sponges also increased the area of new bone formation, but the addition of rhBMP-9 did not enhance osteogenesis. Although chitosan sponges were fully resorbed after 8 weeks and showed osteoconductive properties, they were not effective carriers for rhBMP-9, likely due to weaker adsorption and inability to sustain the factor’s concentration over time. In chitosan groups, newly formed bone was primarily localized at the defect margins, while connective tissue predominated in the center. Collagen sponges are more effective carriers of rhBMP-9, promoting superior defect closure and bone regeneration due to their slower degradation and higher adsorption capacity. Chitosan sponges possess osteoconductive and hemostatic properties, but their limitations as rhBMP-9 carriers make them less suitable in this context. The choice between the two materials should depend on the therapeutic goal: collagen for growth factor-mediated osteoinduction, and chitosan for rapid resorption and hemostasis [107].

Scaffolds proposed as a method for cranial bone regeneration were described by Huang et al. They developed sponges based on TGF-β3, recombinant human-like collagen, and chitosan, loaded with human periodontal ligament stem cells to repair cranial defects in rats. A chitosan sponge was selected due to its optimal properties such as swelling ratio, water absorption, and moisture retention. It exhibited a porous structure, confirmed by SEM. The sponge showed good cellular biocompatibility and underwent complete degradation at the implantation site after 90 days. In vitro tests demonstrated that the sponge supported the proliferation and osteogenic differentiation of stem cells. In a rat cranial defect model, the formulation was shown to promote cranial bone development. Histological results confirmed a greater amount of newly formed bone and fibrous tissue, with no signs of inflammation. The study demonstrated that human periodontal ligament stem cells are effective for cranial injury repair, and TGF-β3 accelerates their osteogenic differentiation. The hybrid sponge represents a promising therapeutic strategy for the regeneration of large cranial defects, offering potential applications in regenerative medicine [108].

Enrichment of biomaterials with bioactive factors is a common practice in tissue engineering. Park et al. developed a chitosan sponge that releases platelet-derived growth factor (PDGF) to improve periodontal bone regeneration. The sponge was produced by lyophilization of a chitosan solution, cross-linking with tripolyphosphate and lyophilization again, resulting in a porous structure with a pore diameter of about 100 μm. SEM analysis confirmed a uniform porous structure suitable for cell migration and growth. Release kinetics showed an initial rapid release of PDGF within the first day, followed by controlled release for 6 days, maintaining therapeutic concentrations for 3 weeks. Tests with rat osteoblasts showed high cell adhesion and proliferation on the sponge with PDGF, with no signs of cytotoxicity. In vivo studies on a cranial defect model in rats showed a significant increase in osteogenesis in the group with PDGF-containing sponge, with rapid calcification and formation of new bone bridges after 4 weeks. The sponge with PDGF showed excellent osteoconductive properties and the ability to release growth factor in a controlled manner, making it a promising material for periodontal bone regeneration, such as in defects after tooth extraction or bone defects [109].

Enriching formulations with nanoparticles is a popular practice in biomaterials engineering. Titanium oxide (TiO_2_) nanoparticles as a sponge additive were used by Ikono et al. The size of the nanoparticles was 20 nm. The hybrid sponge was obtained by freeze-drying a solution of chitosan and nanomaterial. SEM analysis showed an even distribution of TiO_2_ nanoparticles on the surface. The scaffolds showed high porosity and biomineralization ability in simulated body fluid, especially at 50% TiO_2_ content. Bioassays using mouse mesenchymal stem cells showed good cell adhesion and proliferation and no cytotoxicity. Expression of osteogenic genes (DMP1 and OCN) was significantly higher in the group with 50% TiO_2_, indicating increased osteoinductivity. The scaffold with 50% TiO_2_ showed the best mechanical, biomineralization and osteogenic properties, making it a promising material for bone regeneration in complex defects such as tooth extraction defects and cysts. The study underscores the potential of hybridizing chitosan with TiO_2_ in bone tissue engineering which may find application in bone defect reconstruction in dentistry [110]. The addition of TiO_2_ prolonged the integrity of the sponges, indicating that their half-life can be regulated by appropriate additives.

Al-Mofty et al. developed multifunctional hemostatic sponges based on chitosan and poly(vinyl alcohol), enriched with hydroxyapatite and ciprofloxacin, intended for bleeding control, particularly in bone surgery. The sponges were modified with glycerol and citric acid to improve their mechanical properties. The addition of hydroxyapatite supports bone regeneration, while ciprofloxacin provides antibacterial activity against *E. coli*, *P. aeruginosa*, and *S. aureus*. The hybrid sponge demonstrated the most favorable properties: high biocompatibility, good mechanical strength, platelet aggregation capability, and strong antibacterial effects due to the synergy between hydroxyapatite and ciprofloxacin. The sponges exhibited high swelling capacity and rapid degradation (70% mass loss after 7 days). The study indicated that this sponge is a promising material for applications in bone and dental surgery [56].

A hybrid material was described by Lei et al., who developed sponge-like scaffolds based on chitosan and boron-doped silica nanofibers for bone regeneration. These scaffolds, characterized by high porosity and flexibility, were designed to repair irregular bone defects, support skeletal regeneration, and accelerate post-injury healing. The study utilized electrospinning to produce SiO_2_-B nanofibers, which were then homogenized into short fibers and combined with a chitosan solution. Through chemical crosslinking and lyophilization, stable 3D scaffolds were obtained. SiO_2_-B nanofibers support angiogenesis and osteogenesis by releasing silicon (Si^4+^) and boron (B^3+^) ions, while also inhibiting inflammatory responses through modulation of the TLR signaling pathway. Chitosan provides biocompatibility, antibacterial and anti-inflammatory properties, and promotes cell adhesion due to its porous structure and water absorption capacity, which also facilitates hemostasis. The scaffolds were evaluated for their physicochemical, mechanical, and biological properties in vitro, including angiogenesis and coagulation potential. In vivo tests using a rat cranial defect model showed that the scaffolds significantly supported bone regeneration and extracellular matrix deposition. They also demonstrated effective antibacterial activity against both Gram-positive and Gram-negative bacteria and anti-inflammatory effects by promoting macrophage polarization toward the M2 phenotype and reducing oxidative stress [111].

As an interesting highlight, the study by Wang et al. can be mentioned. They developed bioactive composite microsponge scaffolds made from chitosan and nano-hydroxyapatite, intended for the regeneration of large bone defects. The microsponges were fabricated using a microemulsion method with a microfluidic device, followed by a freeze–thaw process in an ethanol/NaOH solution. The resulting microsponges exhibited a porous structure, high porosity, strong swelling capacity, and shape memory properties, making them suitable for filling irregular bone defects. In vitro studies demonstrated good bioactivity of the microsponges—apatite formation was observed in simulated body fluid, indicating their potential for integration with bone tissue. The microsponges also exhibited antibacterial activity against *S. aureus* and *E. coli*, and their biocompatibility was confirmed in cultures of rat bone marrow-derived mesenchymal stem cells. These cells adhered well to the microsponges, proliferated, and showed signs of osteogenic differentiation, as evidenced by increased expression of BMP-2 and Runx2 genes and elevated alkaline phosphatase activity. In vivo, the microsponges were implanted into cranial bone defects in rats without prior cell seeding. After 12 weeks, substantial bone regeneration was observed compared to the control group and the group treated with chitosan alone. Micro-CT analysis revealed a greater volume of newly formed bone, and histological staining confirmed the presence of mineralized bone tissue and expression of osteogenic markers. These results indicate that the microsponges are a promising bone substitute material for tissue engineering, particularly in cases requiring minimally invasive treatment approaches and the filling of irregular bone defects. The authors suggested their potential application in tooth extraction sockets and the maxillary sinus in dental surgery [112].

### 5.4. Chitosan Sponges as Drug Delivery Systems

**Figure 9:**
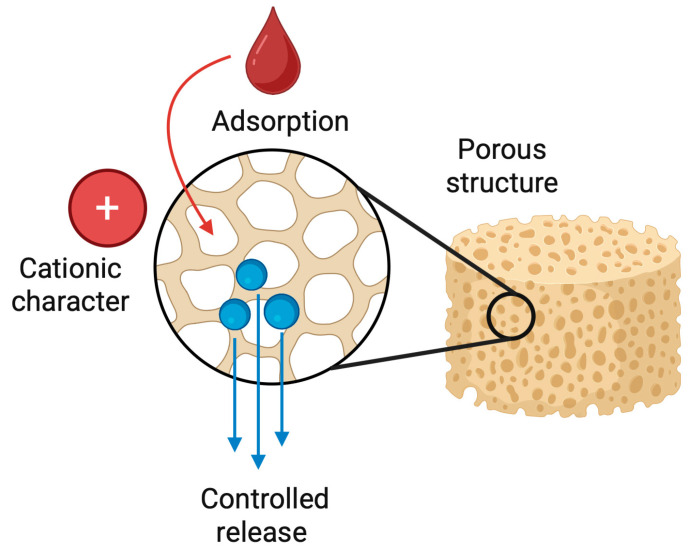
Schematic representation of a chitosan-based sponge as a drug delivery platform. The highly porous structure of the chitosan sponge enables efficient adsorption and entrapment of therapeutic agents within its internal network. Due to the cationic character of chitosan, drugs and bioactive molecules are immobilized through electrostatic interactions and physical retention. Following application to the target site, the sponge gradually releases the loaded therapeutics in a controlled manner, providing sustained local concentrations while maintaining biocompatibility and structural support.

Chitosan-based sponges function not only as structural scaffolds but also as efficient and biocompatible drug delivery platforms. Their highly porous architecture, large internal surface area, and cationic character enable the adsorption, entrapment, and controlled release of a wide range of therapeutic agents [113]. Drugs may be incorporated directly into the polymer matrix during fabrication or introduced through post-loading, while chemical modification, crosslinking density, and pore size allow precise tuning of release kinetics (Figure 9) [114].

The intrinsic mucoadhesive properties of chitosan prolong the residence time of sponges in the oral cavity, facilitating sustained exposure of therapeutics at the target site [7]. This is particularly relevant in periodontal pockets, post-extraction sockets, peri-implant tissues, and infected pulp chambers, where local delivery is preferred over systemic administration. By maintaining high drug concentrations locally and minimizing systemic exposure, chitosan sponges reduce side effects and improve therapeutic efficacy, especially in cases requiring prolonged antimicrobial or anti-inflammatory action [115].

Chitosan sponges have been explored as carriers for antibiotics (e.g., tetracycline, metronidazole, ciprofloxacin), anti-inflammatory drugs, calcium hydroxide, antiseptics, and growth factors such as PDGF, BMPs, or TGF-β3. Several studies have demonstrated their ability to disrupt oral biofilms, inhibit Gram-positive and Gram-negative pathogens, and enhance tissue healing in periodontal and post-surgical environments. Shen et al. prepared tripolyphosphate-crosslinked sponges loaded with tetracycline, achieving sustained release for up to 11 days and prolonged antibacterial activity against periodontopathogens, indicating their usefulness in periodontal pockets [93]. Alghofaily et al. incorporated Augmentin and a modified triple-antibiotic paste into chitosan–gelatin sponges, demonstrating effective eradication of *E. faecalis* biofilms and favorable cytocompatibility with human mesenchymal stem cells, a finding relevant for regenerative endodontic procedures [85].

Beyond antimicrobial therapy, chitosan sponges have been developed for controlled release of osteogenic biologics. Park et al. engineered chitosan sponges crosslinked with tripolyphosphate and loaded with PDGF, achieving initial burst release followed by controlled diffusion for over six days. In a rat cranial defect model, the PDGF-releasing sponges significantly accelerated bone formation and mineralized tissue bridging the defect after four weeks [109]. Similarly, Li et al. incorporated TGF-β3 into lyophilized chitosan scaffolds, which enhanced osteogenic differentiation of periodontal ligament stem cells and extracellular matrix deposition, highlighting the feasibility of chitosan sponges as growth-factor carriers for periodontal regeneration [92].

Nano-engineered sponges further expand therapeutic functionality. The incorporation of titanium oxide nanoparticles into chitosan matrices increased mechanical integrity, antibacterial performance, and osteoinductivity, with significantly elevated expression of osteogenic markers such as DMP-1 and osteocalcin in vitro [116]. Hybrid chitosan–hydroxyapatite sponges loaded with ciprofloxacin demonstrated dual performance: rapid hemostasis and strong antibacterial effects, while simultaneously providing osteoconductive support for bone repair [56].

Depending on the formulation strategy, drug release from chitosan sponges may follow burst-release, sustained-release, or biphasic profiles. Ionic crosslinking, composite blending with hydroxyapatite or alginate, lyophilization parameters, and nanoparticle incorporation enable precise control of degradation rate and therapeutic delivery. These multifunctional systems allow simultaneous control of infection, inflammation, hemostasis, and tissue regeneration. In clinical translation, such properties are advantageous in complex dental scenarios, including deep periodontal pockets, infected extraction sites, implant-associated defects, and pulp-tissue regeneration.

Current evidence confirms that chitosan-based sponges are versatile and tunable drug delivery systems, capable of localized, sustained, and bioactive therapeutic release. Their ability to couple pharmacological delivery with structural support, hemostasis, and intrinsic antimicrobial activity positions chitosan sponges as next-generation biomaterials for targeted dental therapies and broader regenerative medicine.

## 6. Future Perspectives and Clinical Translation

**Figure 10:**
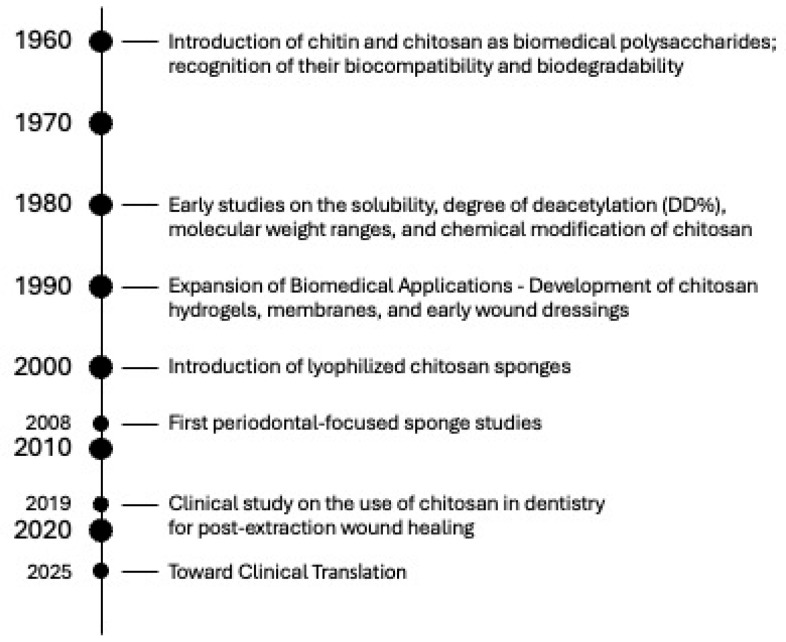
Timeline of Key Milestones in the Development of Chitosan Sponges for Dental Applications.

Over the past several decades, the development of chitosan-based biomaterials has evolved from fundamental studies on chitin chemistry to the creation of advanced, multifunctional scaffolds for dental and regenerative applications. This progression reflects continuous improvements in extraction methods, structural modification, fabrication techniques, and biological understanding. To contextualize the current challenges and opportunities in clinical translation, Figure 10 presents a timeline summarizing the key milestones in the evolution of chitosan research, from the initial recognition of its biocompatibility, through the emergence of lyophilized sponges and early periodontal applications, to recent advancements in smart nanocomposites and drug-releasing systems.

The translation of chitosan-based sponges from bench to bedside represents a promising yet challenging frontier in regenerative dentistry. Although numerous preclinical studies have confirmed their biocompatibility, hemostatic properties, and regenerative potential, clinical implementation remains limited. A major obstacle lies in the lack of standardization in chitosan extraction, purification, and sponge fabrication processes. Variations in the degree of deacetylation, molecular weight, crosslinking density, and pore size significantly affect degradation rate, mechanical strength, and bioactivity, making direct comparison across studies difficult. Establishing uniform quality control criteria and regulatory standards will be essential to ensure reproducible biological performance and safety. Because chitosan is derived from natural marine biomass, its physicochemical properties are inherently influenced by the biological variability of its raw material. Differences in species, habitat, seasonal factors, and extraction conditions lead to fluctuations in protein residues, mineral content, and acetylation patterns, all of which significantly affect downstream processing and the final performance of the sponge. This intrinsic variability poses additional challenges in achieving regulatory-grade standardization compared with synthetic or mammalian collagen-based scaffolds.

Despite these manufacturing challenges, current research must also address several specific and measurable performance gaps that limit the reproducibility and predictability of chitosan sponge-based therapies. One critical issue is the lack of controlled biodegradation profiles, as existing formulations degrade over widely variable timescales, from a few days to several weeks, while clinically relevant applications require a predictable window of resorption, such as approximately 1–2 weeks for extraction sockets or 2–4 weeks for periodontal defects. Similarly, many chitosan sponges exhibit insufficient mechanical integrity, particularly after hydration; to withstand manipulation and intraoral forces, scaffolds should achieve compressive moduli in the range of 20–30 kPa, a threshold not consistently met by current materials. Drug-loaded systems also suffer from excessive initial burst release, often exceeding 50% of the drug payload, which compromises sustained therapeutic activity; achieving stable antimicrobial or regenerative factor release for 7–10 days remains a key unmet requirement. Furthermore, high swelling ratios, occasionally surpassing 500–1000%, lead to loss of dimensional stability and compromised retention in periodontal pockets, whereas optimal clinical performance would require swelling to remain below 200–300%. Finally, the pore architecture of many sponges remains poorly controlled, with broad and non-uniform pore size distributions; effective cellular infiltration and tissue integration demand a more standardized network with pore diameters in the 100–300 μm range. The bioresorption rate of chitosan is one of the most inconsistently reported parameters, with degradation times ranging from several months to over a year. Such variability reflects differences in molecular weight, degree of deacetylation, crystallinity, crosslinking, and fabrication methods, as well as local enzymatic activity. In dental surgery, however, prolonged degradation is generally undesirable, as post-extraction sockets, periodontal wounds, and regenerative sites typically require materials that resorb within 1–4 weeks to avoid foreign-body persistence and interference with tissue remodeling. Excessively slow-resorbing chitosan structures may impair healing or trigger chronic inflammatory responses. Addressing these quantifiable gaps through advanced material engineering and standardized characterization protocols will be essential for the development of next-generation chitosan sponges capable of reliable clinical translation (Table 3).

In addition to material variability, the regulatory landscape further limits the successful translation of chitosan sponges into clinical practice. The absence of harmonized GMP-compliant production guidelines for chitosan-based medical devices complicates the establishment of reproducible manufacturing workflows, particularly because chitosan’s physicochemical properties depend strongly on its biological source and processing conditions. For clinical approval, manufacturers must meet stringent requirements related to raw material traceability, endotoxin control, sterility assurance, and long-term biocompatibility, all of which remain insufficiently standardized for chitosan. Moreover, obtaining CE marking under the European Medical Device Regulation (MDR 2017/745) requires extensive documentation of safety, stability, and clinical performance, a process hindered by the current lack of reference specifications for natural polymer devices. Similarly, in the United States, the FDA classification of chitosan sponges typically falls under Class II medical devices, yet many advanced formulations lack a clear predicate device, potentially necessitating a more complex De Novo pathway. These regulatory uncertainties, combined with the need for robust clinical evidence, create significant barriers to commercialization and delay broader clinical adoption. Consequently, the development of unified material standards, GMP protocols, and clear regulatory guidance will be crucial to accelerating the safe and effective integration of chitosan sponges into routine dental practice.

Another critical step toward clinical translation involves conducting well-designed randomized clinical trials. Most available data originate from small-scale or animal studies, which do not fully represent the complexity of human oral tissues. Clinical trials assessing chitosan sponge dressings in post-extraction sockets, periodontal defects, and alveolar bone regeneration should include long-term follow-up to evaluate biodegradation, inflammatory response, and tissue integration. Moreover, the incorporation of biomarkers of healing and bone remodeling may provide deeper mechanistic insights into their clinical performance.

Future development of chitosan sponges will likely focus on multifunctional and “smart” systems capable of controlled degradation and targeted release of therapeutic agents. The combination of chitosan with growth factors (e.g., BMPs, PDGF, VEGF), stem cells, or nanoparticles offers opportunities for personalized, precision-based regenerative approaches. Incorporating stimuli-responsive polymers and bioactive nanostructures could further enhance local drug delivery, antimicrobial efficacy, and angiogenesis. The emergence of 3D printing and bioprinting techniques also opens new avenues for fabricating patient-specific chitosan scaffolds tailored to defect morphology.

**Figure 11:**
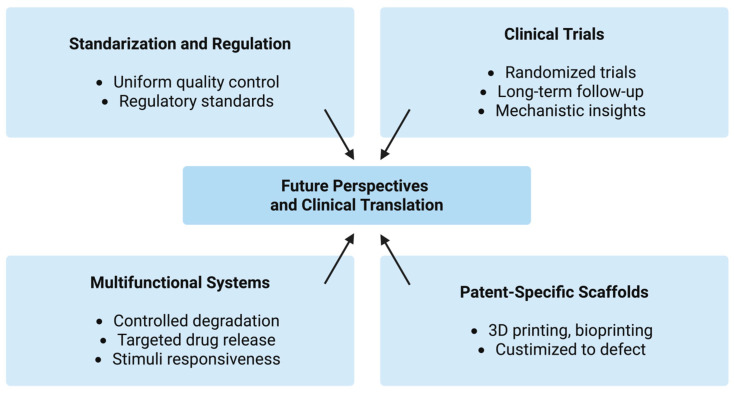
Future perspectives and clinical translation pathways of chitosan-based sponges in regenerative dentistry.

From a clinical perspective, the cost-effectiveness, ease of application, and favorable safety profile of chitosan make it an attractive alternative to conventional collagen (higher production costs, the need for complex purification from mammalian tissues, and the potential, although low, risk of immunogenicity or disease transmission) or synthetic materials. Its use as an adjunct in periodontal and implant therapy, as well as in pediatric and oral surgery, could reduce healing time and postoperative complications. However, regulatory approval will depend on rigorous toxicological assessment, manufacturing reproducibility, and compliance with Good Manufacturing Practice GMP standards (Figure 11).

## 7. Chitosan Sponges Beyond Dentistry: Cross-Disciplinary Biomedical Applications

**Figure 12:**
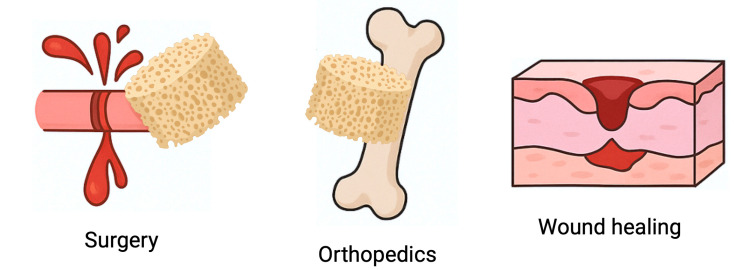
Chitosan sponges beyond dentistry: cross-disciplinary biomedical applications. Schematic illustration of the versatility of chitosan-based sponges in different medical fields. Owing to their high porosity, liquid-absorption capacity, mucoadhesive properties and intrinsic antibacterial activity, these sponges are used as hemostatic dressings in general surgery, wound-healing materials in dermatology and trauma care, and osteoconductive scaffolds supporting bone repair in orthopedics. Their ability to stabilize blood clots, protect the defect site and promote tissue regeneration demonstrates that chitosan sponges function as a broadly applicable platform in regenerative medicine beyond the oral cavity.

Although this review focuses on dental medicine, chitosan-based sponges have been increasingly investigated across multiple biomedical fields. Their ability to absorb large volumes of physiological fluids, support blood clot formation, adhere to wet tissues, and degrade in a controlled manner makes them attractive hemostatic and regenerative dressings in general surgery, dermatology, and orthopedics (Figure 12). In trauma surgery, chitosan sponges are used to rapidly control hemorrhage and stabilize clots, outperforming gauze and several synthetic dressings due to their intrinsic pro-coagulant properties and bioresorbability [117,118]. In chronic wound care, such as diabetic ulcers or burns, chitosan sponges promote granulation tissue formation, reduce microbial load, maintain a moist environment, and accelerate re-epithelialization, often outperforming traditional collagen or alginate dressings [52].

Bone-related applications extend beyond the maxillofacial region. Composite chitosan sponges combined with hydroxyapatite, calcium phosphates, or bioactive nanoparticles have shown promising outcomes in the treatment of long-bone defects, cranial injuries, and spinal fusion models, where they act as osteoconductive scaffolds promoting angiogenesis and mineralization [119]. In addition, their ability to incorporate and release growth factors, stem cells, and therapeutic molecules has led to experimental use in cartilage regeneration, peripheral nerve repair, and anticancer drug delivery [120]. These studies demonstrate that chitosan sponges are not limited to the oral cavity but represent a broad platform technology with translational potential across multiple medical specialties.

This broader biomedical relevance strengthens their position as next-generation natural polymer sponges that combine hemostatic activity, local drug delivery, and regenerative functionality. Understanding these wider applications may help accelerate the clinical translation of chitosan sponges in dentistry, as safety, manufacturing, and regulatory frameworks developed in other fields can support their approval and use in oral medicine.

## 8. Conclusions

Chitosan-based sponges represent a highly promising biomaterial platform in regenerative dentistry, combining biological functionality with structural versatility. Their three-dimensional porous network allows efficient fluid absorption, gas exchange, and nutrient diffusion, conditions vital for tissue regeneration. The cationic nature of chitosan promotes platelet activation and rapid hemostasis, while its antibacterial and anti-inflammatory properties support wound healing and reduce infection risk in the oral cavity.

Beyond serving as hemostatic dressings, chitosan sponges function as biodegradable scaffolds that facilitate cell adhesion, proliferation, and differentiation. When loaded with osteogenic or angiogenic agents such as BMPs, VEGF, or calcium phosphates, they can effectively enhance bone regeneration and vascularization, making them suitable for alveolar repair, periodontal therapy, and implant site preservation. Their physicochemical properties, including pore size, deacetylation degree, and crosslinking density, can be optimized to control mechanical strength, degradation rate, and drug release behavior.

Despite these advantages, clinical translation remains limited due to variations in material processing and the lack of standardized evaluation methods. Future research should emphasize GMP-compliant production, standardized characterization, and long-term clinical trials to ensure safety and reproducibility. With continued interdisciplinary collaboration, chitosan sponges are poised to become clinically validated, customizable biomaterials for next-generation regenerative dental therapies.

## Figures and Tables

**Table 1:** Summary of physicochemical properties of chitosan.

Property	Influence Factor	Biological Relevance
Degree of deacetylation	↑ DD → ↑ cationic charge	Enhances hemostasis, mucoadhesion
Molecular weight	↑ MW → ↓ solubility	Affects degradation rate
Crosslinking density	Higher → ↑ mechanical strength	Slower biodegradation
Pore size	50–200 μm optimal	Promotes cell migration

↑—increase; →—results in; ↓—decrease.

**Table 2:** Overview of fabrication methods for chitosan sponge.

Method	Description	Advantages	Limitations
Freeze-drying	Freezing & sublimation	High porosity	Time-consuming
Foaming	Mechanical or chemical air entrapment	Easy control of pore size	Lower uniformity
Crosslinking	Genipin, glutaraldehyde	Improved stability	Possible cytotoxicity
Composite blending	With collagen, gelatin, HA	Enhanced bioactivity	Complex formulation

**Table 3:** Key design and performance guidelines for next-generation chitosan sponges in dental applications.

Parameter	Observed Limitations	Recommended Target	Rational
Biodegradation rate	Highly variable, from days to >1 year	1–2 weeks for extraction sockets; 2–4 weeks for periodontal defects	Prevents prolonged foreign-body presence; aligns with healing dynamics
Mechanical integrity (after hydration)	Insufficient stiffness; collapse under intraoral forces	Compressive modulus 20–30 kPa	Required to withstand manipulation, irrigation and chewing forces
Drug release profile	High burst release (>50% payload)	Sustained release for 7–10 days, minimal burst	Maintains antimicrobial/regenerative efficacy; reduces reinfection risk
Swelling ratio	Very high (500–1000%), loss of dimensional stability	<200–300%	Ensures pocket retention and shape stability
Pore size and architecture	Broad, non-uniform distributions	Uniform, interconnected pores 100–300 μm	Optimal for cell infiltration, angiogenesis, and tissue regeneration
Crosslinking control	Variable stability and degradation	Tunable ionic/covalent crosslinking	Allows fine control of stiffness and resorption
Molecular variability (MW, DD%)	Inconsistent physicochemical properties	Standardized ranges for MW & DD% across studies	Ensures reproducible biological performance

## Data Availability

No new data were created or analyzed in this study.
